# *Stenotrophomonas maltophilia*, a Pathogen of Increasing Relevance to Dermatologists: A Case Report and Review of the Literature

**DOI:** 10.3390/antibiotics11101398

**Published:** 2022-10-12

**Authors:** Annika Belzer, Emma Weiss, Farshid Etaee, Christopher G. Bunick, William Damsky, Caroline A. Nelson

**Affiliations:** 1 Yale School of Medicine, New Haven, CT 06510, USA; 2 Department of Dermatology, Yale School of Medicine, New Haven, CT 06510, USA; 3 Department of Internal Medicine, Yale School of Medicine, New Haven, CT 06510, USA; 4 Program in Translational Biomedicine, Yale School of Medicine, New Haven, CT 06510, USA

**Keywords:** Gram-negative bacteria, nosocomial infection, multidrug-resistant, skin and soft tissue infection, *Stenotrophomonas maltophilia*

## Abstract

*Stenotrophomonas maltophilia* is a Gram-negative bacillus that causes skin and soft tissue infections (SSTI), as well as bacteremia, pneumonia, and urinary tract infections. *S. maltophilia* infections are typically nosocomial and are often transmitted through water sources. Although historically described in immunocompromised hosts, *S. maltophilia* prevalence is increasing in both immunocompromised and immunocompetent populations. In light of high morbidity and mortality, it is critical that dermatologists are aware of this organism because of the limited options for therapy. Here, we describe a case of a *S. maltophilia* abscess with bacteremia in a patient with chronic lymphocytic leukemia and aplastic anemia that was successfully treated with trimethoprim–sulfamethoxazole. We also review the current standard of care and propose an algorithm for the treatment of *S. maltophilia* infection.

## 1. Introduction

*Stenotrophomonas maltophilia* is a non-fermentative Gram-negative bacillus that can be transmitted by nosocomial sources including contaminated disinfectants, intravenous fluids, hospital water, catheters, central venous and arterial pressure monitors, dialysis equipment, blood gas analyzers, ventilation circuits, thermometers, and intra-aortic balloon pumps [1,2]. Although *S. maltophilia* is known to colonize the skin, gastrointestinal tract, and respiratory tract, *S. maltophilia* can cause opportunistic infections, including bacteremia, pulmonary infections, urinary tract infections, and skin and soft tissue infections (SSTI), in immunocompromised hosts [3,4]. Meningitis and endocarditis have also been reported in rare cases [3]. *S. maltophilia* infection is associated with a high morbidity and mortality, with a systematic review reporting an attributable mortality rate of 37.5% [5]. Mortality has been associated with host factors, including an Acute Physiology and Chronic Health Evaluation (APACHE) score greater than 15 and a Sequential Organ Failure Assessment (SOFA) score greater than 6 [6,7]. Intensive care unit (ICU) admission and delay in treatment, in part due to resistance to commonly employed antibiotic classes, have also been associated with mortality [8].

Risk factors for *S. maltophilia* infection include prolonged hospitalization and ICU admission, malignancy including leukemia and lymphoma, neutropenia, mechanical ventilation for greater than seven days or tracheostomy, central venous catheter (CVC) or other foreign bodies, mucosal damage such as that which occurs with chemotherapy or radiation, and diarrhea [1,3]. In a retrospective study of *S. maltophilia* infections within a single hospital system from 2005 to 2010 (*n* = 68), 66.2% of patients were immunocompromised; 68.9% of the immunocompromised cohort was being treated with a systemic corticosteroid [9], 41.2% of patients had a CVC and 47.1% had an indwelling catheter other than a CVC [9]. Exposure to broad-spectrum antibiotics such as carbapenems, extended-spectrum cephalosporins, and fluoroquinolones is also a significant risk factor for *S. maltophilia* infection in the setting of intrinsic resistance and selective pressure [1,10]. In a matched case-control study, prior imipenem therapy was ten times more frequent in *S. maltophilia* cases versus the controls [11]. It is thought that this is due, in part, to *S. maltophilia* overgrowth, as it is resistant to carbapenems [3].

SSTI comprise up to 15% of *S. maltophilia* infections, the source of which can be primary inoculation or bacteremia [1]. *S. maltophilia* SSTI has a broad range of potential presentations, including primary or metastatic cellulitis, abscess, multifocal purpura, and ulcer or ecthyma gangrenosum [1]. Potentially life-threatening deep infections including fasciitis and myositis have also been reported [1]. Here, we highlight a unique case of SSTI caused by *S. maltophilia* that was initially misdiagnosed as *Pseudomonas aeruginosa* on superficial culture and demonstrated resistance to several antibiotics.

## 2. Case Report

A 77-year-old man with chronic lymphocytic leukemia (CLL) and aplastic anemia was admitted for neutropenic fever and a lesion in the right groin. Two weeks prior, he had been admitted with an abscess in the same area, and a superficial culture grew *P. aeruginosa*. Based on susceptibility testing, he was treated with 4.5 g intravenous piperacillin–tazobactam every six hours for five days. After remaining afebrile with an otherwise negative infectious work up, piperacillin–tazobactam was transitioned to ciprofloxacin. He was discharged on 500 mg oral ciprofloxacin twice daily and 875–125 mg oral amoxicillin–clavulanate twice daily with a plan to continue indefinitely for antibacterial prophylaxis. Despite antimicrobial therapy, the lesion worsened and ultimately ulcerated. 

**Figure 1:**
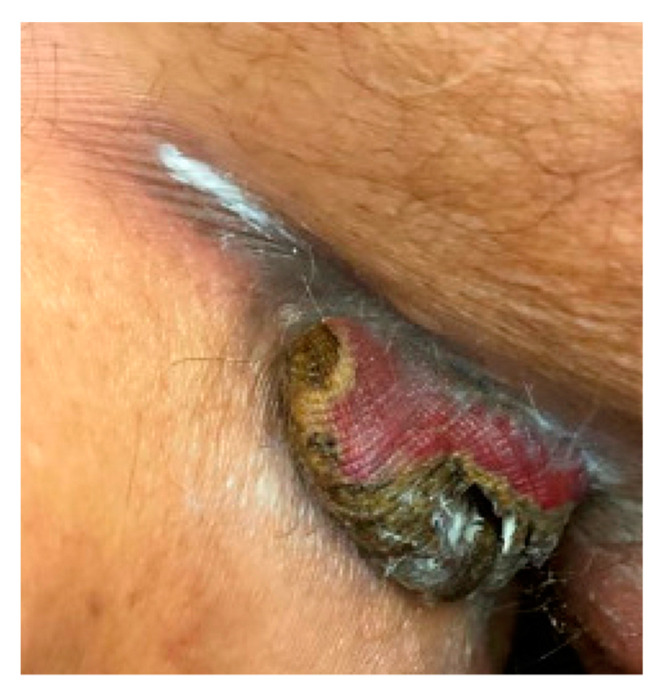
Dermatologic examination demonstrated a purple-red indurated nodule with central ulceration and yellow crust in the right groin.

**Figure 2:**
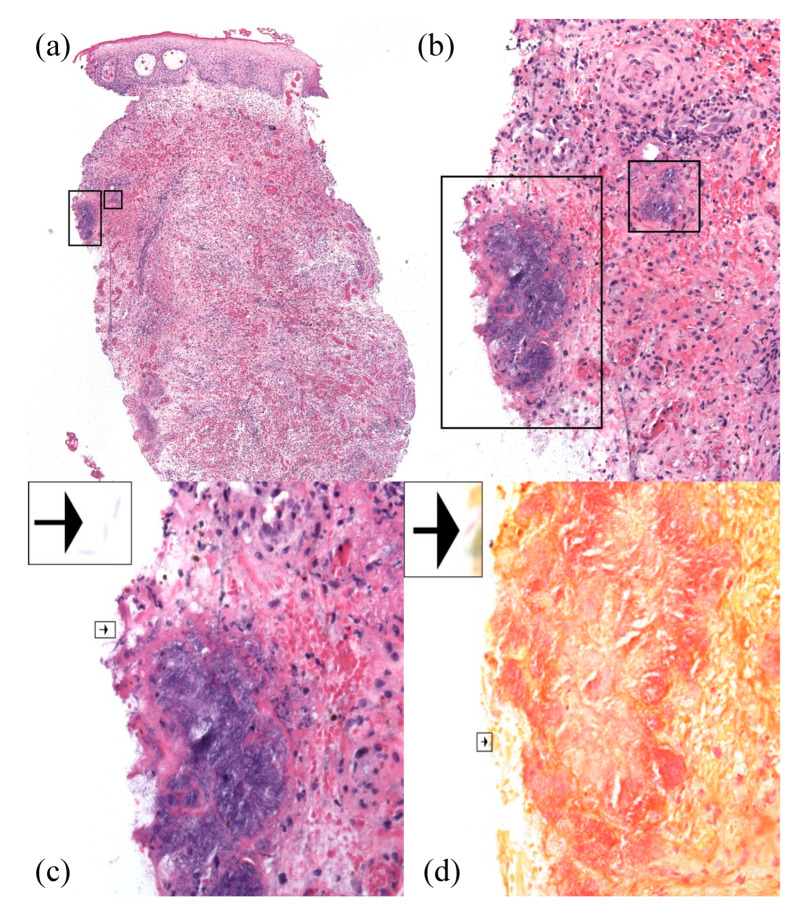
Histopathology (hematoxylin and eosin) demonstrated numerous organisms with a rod to focally filamentous morphology, as well as edema, dilated blood vessels, extravasated erythrocytes, and mixed inflammatory infiltrate at 40× (**a**), 200× (**b**), and 400× (**c**). Gram stain confirmed the presence of Gram-negative organisms, consistent with *S. maltophilia* (**d**, 400×). Individual organisms were visualized at the edge of the tissue specimen (arrows).

Examination at the time of readmission showed a purple-red indurated nodule with central ulceration and yellow crust in the right groin (Figure 1). Serum analyses demonstrated a white blood cell count of 400 cells/mL and an absolute neutrophil count of zero. Procalcitonin and lactate were within the normal limits. Two 3mm punch biopsies were performed for histopathological examination and sterile tissue culture. Histopathologic examination demonstrated edema, superficial and deep mixed inflammatory infiltrate, and numerous organisms with a rod to focally filamentous morphology (Figure 2a–c). The organisms were Gram-negative, ruling out *Staphylococcus aureus*, but potentially consistent with the previous diagnosis of *P. aeruginosa* abscess (Figure 2d). *S. maltophilia* was identified with a sterile tissue culture. *S. maltophilia* susceptible to trimethoprim–sulfamethoxazole and minocycline was also identified with blood cultures. Susceptibility testing for other antibiotics was not performed. Taken together, these findings were consistent with a diagnosis of *S. maltophilia* SSTI.

**Figure 3:**
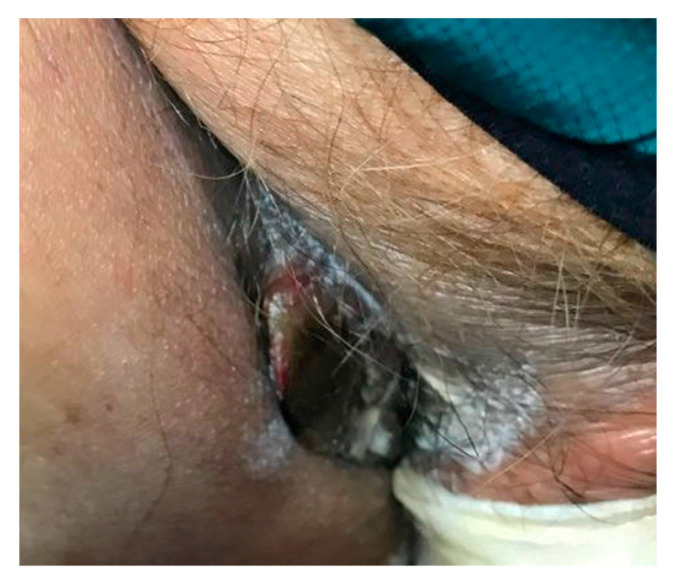
At follow up, the nodule had decreased in size and induration with interval improvement of the erythema, ulceration, and crust.

The patient was treated with two tablets of 800–160 mg oral trimethoprim–sulfamethoxazole every eight hours, along with intravenous piperacillin–tazobactam in the setting of prior culture demonstrating *P. aeruginosa*. There was concern for inadequate antibiotic penetration due to necrosis and a resulting decrease in tissue perfusion, leading to the consideration of excision [12]. However, debridement was deferred due to thrombocytopenia. At four days of treatment, trimethoprim–sulfamethoxazole was transitioned to 100 mg oral minocycline twice daily in the setting of severe pancytopenia, along with continued oral ciprofloxacin and amoxicillin–clavulanate, with a plan to continue for 30 days following discharge. At the two-month follow-up, the patient remained afebrile. The lesion had decreased in size with interval improvement of the induration, ulceration, erythema, and crust, despite persistent necrosis (Figure 3).

## 3. Discussion

*S. maltophilia* infections are increasing in prevalence; this is critical to outpatient and inpatient care because of the high morbidity and mortality. A series of studies performed at a single hospital center in Houston, Texas, from 1986 to 2002 demonstrated an increase in *S. maltophilia* from 2% of all Gram-negative rod isolates from patients with a cancer diagnosis, to 7%, bringing *S. maltophilia* from the ninth to the fifth most commonly isolated Gram-negative organism [3,13]. A greater severity of *S. maltophilia* infections in patients with a cancer diagnosis was also reported, with a significant increase in cases of moderate to high-grade *S. maltophilia* bacteremia from 4% in 1998 to 17% in 2004 [3,13]. Although classically described in immunocompromised hosts, *S. maltophilia* is now being observed in immunocompetent patients without known risk factors in the setting of community transmission [1,14]. It is thought that the increase in the prevalence of *S. maltophilia* infections is, in part, due to the increased use of broad-spectrum antibiotics such as carbapenems, advances in oncologic therapy leading to a great number of susceptible hosts, and the use of indwelling catheters and devices [1]. However, research on the incidence of *S. maltophilia* is sparse, and further investigation is warranted in light of this organism’s increasing relevance to both outpatient and inpatient dermatologists.

**Figure 4:**
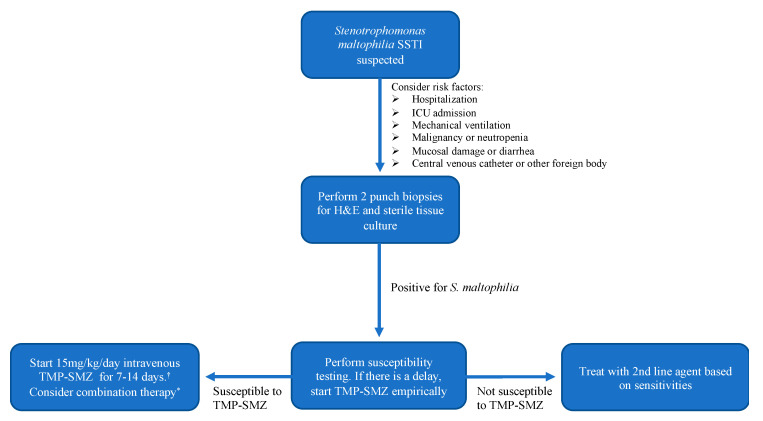
Diagnostic and treatment algorithm for the patient with suspected *S. maltophilia* SSTI. ^†^ In the case of sulfonamide allergy, desensitization may be pursued. If this is not possible, treatment with levofloxacin or minocycline should be initiated if the culture demonstrates sensitivity. * Consider combination therapy such as trimethoprim–sulfamethoxazole plus a third-generation cephalosporin or extended-spectrum penicillin [15].

Treatment of *S. maltophilia* is complex, as this multi-drug resistant organism is not susceptible to commonly employed antibiotic classes including beta-lactams and aminoglycosides (Figure 4) [1]. The standard of care for *S. maltophilia* infection is high dose trimethoprim–sulfamethoxazole; studies have reported dosing at 15 mg/kg per day for a seven to fourteen-day course [1]. In patients with an allergy to sulfonamides, desensitization has been suggested [3]. For patients in whom this is not possible, levofloxacin or minocycline are first line if the bacterial culture demonstrates sensitivity. Combination therapy of trimethoprim–sulfamethoxazole with ceftazidime, ticarcillin–clavulanate, minocycline, tigecycline, moxifloxacin, or chloramphenicol has been suggested due to the high resistance rates; however, the definitive benefit of combination therapy has not been convincingly demonstrated in the literature [1]. 

Unfortunately, there is a trend of increasing *S. maltophilia* resistance to the few antibiotics that have been classically efficacious [16]. A 2005 review of bacterial isolates collected through the SENTRY Antimicrobial Surveillance Program from 1997 to 2003 demonstrated 95.5% susceptibility to trimethoprim–sulfamethoxazole [17]. Seven years later, a retrospective study of *S. maltophilia* infection (*n* = 68) reported 85.3% susceptibility to trimethoprim–sulfamethoxazole [9]. This study demonstrated more than 85% susceptibility with colistin (91.2%) and netilmicin (85.3%) as well [9]. In light of increasing resistance, further research into alternative antimicrobial treatment approaches is critical. 

## 4. Conclusions

*S. maltophilia* infections, including SSTI, are increasing in prevalence among both immunocompromised and immunocompetent patients. Prompt diagnosis is critical, as *S. maltophilia* infection does not respond to the majority of antibiotics. In the setting of treatment-resistant SSTI, sterile tissue culture may be warranted to evaluate for the possibility of *S. maltophilia* SSTI. It is critical that dermatologists are aware of this potentially lethal infectious agent so appropriate steps can be taken to assess for *S. maltophilia* antimicrobial resistance and expeditiously initiate appropriate antimicrobial therapy.

## Abbreviations

Skin and soft tissue infection	SSTI
Intensive care unit	ICU
Hematoxylin and eosin stain	H&E
Trimethoprim-sulfamethoxazole	TMP-SMZ
